# Flubendazole as a macrofilaricide: History and background

**DOI:** 10.1371/journal.pntd.0006436

**Published:** 2019-01-16

**Authors:** Timothy G. Geary, Charles D. Mackenzie, Steven A. Silber

**Affiliations:** 1 Institute of Parasitology, McGill University, Ste-Anne-de-Bellevue, Québec, Canada; 2 NTDSC/MDP, Task Force for Global Health, Decatur, Georgia, United States of America; 3 Johnson & Johnson Global Public Health, Janssen Research and Development, LLC., New Brunswick, New Jersey, United States of America; New York Blood Center, UNITED STATES

## Abstract

Benzimidazole anthelmintics have long been employed for the control of soil-transmitted helminth infections. Flubendazole (FBZ) was approved in 1980 for the treatment of gastrointestinal nematode infections in both veterinary and human medicine. It has also long been known that parenteral administration of FBZ can lead to high macrofilaricidal efficacy in a variety of preclinical models and in humans. As part of an effort to stimulate the discovery and development of new macrofilaricides, particularly for onchocerciasis, research has recently been devoted to the development of new formulations that would afford high oral bioavailability of FBZ, paving the way for potential clinical development of this repurposed drug for the treatment of human filariases. This review summarizes the background information that led to this program and summarizes some of the lessons learned from it.

Elimination of the human filarial diseases lymphatic filariasis (LF) and onchocerciasis is a formal objective of the global health community [1,2]. Decades-long control programs leading to elimination rely primarily on annual or semiannual mass drug administration (MDA) campaigns using drugs that kill the microfilarial stages of *Onchocerca volvulus* and *Wuchereria bancrofti*, the primary causative agents of these diseases; the chemotherapeutic regimens also induce prolonged suppression of microfilarial production by female parasites. The long-lasting reduction of the microfilarial burden effectively prevents transmission and, in onchocerciasis patients, the development of dermal and ocular pathology. Elimination can be attained if transmission is blocked until all adult worms in a locale have died (5 to 15 years). The drugs used in MDA campaigns include ivermectin in onchocerciasis areas and diethylcarbamazine in areas in which only LF is present; both agents are combined with albendazole for use in LF control. The use of ivermectin or diethylcarbamazine in areas coendemic with *Loa loa* is problematic due to the danger of severe adverse neurologic sequelae in patients harboring high loads of *L*. *loa* microfilariae [3,4].

Because the regimens used for MDA are not rapidly macrofilaricidal, there is general agreement that availability of a macrofilaricide would significantly accelerate progress toward elimination of filariases, in particular onchocerciasis [5–7]. Macrofilaricidal regimens compatible with the current requirements of MDA programs (brief duration of therapy, high efficacy, very high safety, slow or no microfilaricidal activity) are preferred, but less stringent criteria could be applied to drugs used in a “test and treat” paradigm in which only infected individuals are treated. This paradigm is of particular relevance for the achievement of eradication of these pathogens at the end-stage of MDA programs, when relatively few people would require treatment and for whom a decade-long annual distribution scheme is unlikely to be cost-effective.

Antibiotics that deplete the *Wolbachia* symbiont from filariae, most notably doxycycline, can kill adult parasites after an extended course of therapy [8,9]; proof-of-concept that this regimen could be used in a field setting has been obtained, but adoption of this agent as a primary strategy to attain elimination is incompatible with generally accepted target product profiles (TPPs) for a new macrofilaricide. Recently, the combination of albendazole + ivermectin + diethylcarbamazine was shown to have macrofilaricidal activity against *W*. *bancrofti* after a single treatment [10,11]. If this finding can be confirmed in other areas, it would constitute a major breakthrough for the elimination of LF. However, it is not known whether similar results could be obtained in onchocerciasis, and the inclusion of diethylcarbamazine in a regimen intended for broad use in onchocerciasis regions is not without significant safety concerns. Against this background, there has been a recent resurgence of interest in the discovery and development of new macrofilaricides [5–7]. From these efforts, several agents have been proposed for preclinical or clinical development, including new antibiotics or antibiotic combinations, moxidectin, auranofin, emodepside, imatinib, and other kinase inhibitors [5–7, 12–15].

The case for consideration of flubendazole (FBZ) as a potential macrofilaricide for human use in campaigns against onchocerciasis and LF was outlined in 2011 [16]. To briefly summarize, experiments sponsored by WHO in the 1970s identified FBZ administered parenterally as a highly effective macrofilaricide in multiple animal models. It was found in a small number of pharmacokinetic (PK) experiments that subcutaneous (s.c.) or intramuscular (i.m.) injections provided a depot effect, such that very low plasma levels of the drug were maintained for weeks or months. The drug is licensed and marketed for veterinary and human use for the treatment of gastrointestinal nematode infections in an oral formulation that provides exceptionally limited oral bioavailability, a limiting factor for wide-spread use for filarial diseases. A human trial in Central America in the early 1980s using a parenteral formulation of FBZ (5 weekly intramuscular injections of 750 mg in an unknown vehicle) produced 100% macrofilaricidal efficacy against *O*. *volvulus*. However, this formulation and dosing schedule led to abscess formation at the injection site [17]. At about the same time, a putative prodrug of FBZ, UMF-078, was advanced into preclinical evaluation. Although the compound was efficacious as a macrofilaricide when dosed i.m. in cattle infected with *Onchocerca ochengi* [18], neurotoxicity occurred after two doses. Interpretation of these results is complicated by the fact that, at least in dogs, very little FBZ is detected in plasma compared to the parental UMF-078 (ratio 1:100) [19], and no comparative toxicology studies were done. Because even high doses of FBZ failed to generate neurotoxicity in the studies reported here, the conservative conclusion is that the effects observed with UMF-078 were due to the parent compound and not to the small amount of FBZ produced. Further work on the development of FBZ for this indication was stopped, at least in part due to the availability of ivermectin for onchocerciasis control.

New interest in macrofilaricides, particularly for the elimination of onchocerciasis, prompted a reevaluation of the potential of FBZ for this purpose [16]. As noted, experiments in animal models and in a human trial proved that parenteral administration of FBZ could attain 100% efficacy as a macrofilaricide against model and target pathogens. Because parenteral administration is not compatible for MDA programs, the initial goal was to develop a new formulation of FBZ that provided sufficiently high oral bioavailability to achieve high macrofilaricidal efficacy with a short dosing regimen. In other words, could the high efficacy observed with a low and prolonged exposure profile from parenteral dosing be duplicated with a high and short PK profile attained with a new oral formulation?

The project began by assembling and analyzing available information on the toxicology of FBZ in the public domain and other repositories, including data developed at WHO and during the development of the drug for veterinary registration. The purpose of this exercise was to uncover data that would preclude attempts to reformulate FBZ to enable oral dosing. As a member of the benzimidazole anthelmintic class, FBZ was known to be a tubulin inhibitor [16], and it was anticipated that effects on cell division and development would be evident in vitro and in high-dose animal studies. Indeed, aneugenic and embryotoxic effects similar to those observed with albendazole and other benzimidazole anthelmintics have been reported for FBZ. However, given the potential benefits of a well-tolerated macrofilaricide, these were not thought to present an unsurmountable barrier to human use, especially if a low dose and brief duration of administration could be achieved. In addition, if used in a “test and treat” paradigm (as opposed to MDA), patients could be carefully monitored for evidence of toxicity and dosing modified based on risk–benefit considerations.

This analysis triggered a decision by the Bill and Melinda Gates Foundation (BMGF) to fund preclinical development work with FBZ under the auspices of the Drugs for Neglected Diseases initiative (DND*i*), which led to initial studies with candidate formulations to demonstrate proof-of-concept [18] and then to the development of an amorphous solid dispersion formulation by Abbott Laboratories (later AbbVie) that afforded very high bioavailability and was used for initial toxicological investigations [19, 20]. This formulation also enabled efficacy studies in small animal models of filariases; these data supported the original hypothesis that 5 to 7 day regimens of orally bioavailable FBZ could attain high macrofilaricidal efficacy.

In early 2012, commensurate with its committments to the London Declaration on Neglected Tropical Diseases (http://unitingtocombatntds.org/london-declaration-neglected-tropical-diseases), Janssen Research and Development, LLC (JRD; a subsidiary of Johnson & Johnson, Inc.), the company that had discovered and developed FBZ for human and veterinary use, assumed responsibility for the preclinical and possible clinical development of FBZ. A new stable high-concentration formulation of FBZ developed by JRD [21] was used to conduct more extensive safety, PK/metabolism, and efficacy studies; the results of which are presented in papers appearing in this issue [22–25]. Interest in FBZ as a macrofilaricide also generated studies with target parasites to develop a better understanding of the pharmacological basis for activity, especially the exposure profile needed to cause irreversible damage [26–29]. These studies revealed that the actions of FBZ are consistent with tubulin inhibition and that brief exposures could seriously damage the parasites; however, in an unexpected result, this acute damage was found to be reversible [27].

During the execution of the preclinical program for FBZ, a global consortium of experts from academia, JRD and other pharmaceutical companies, and BMGF proposed a TPP for a new macrofilaricide. Although the TPP evolved as new data accumulated and the logistics of implementing an MDA program were considered, a consensus emerged that oral administration was a priority and that the treatment regimen should be no longer than 10 daily doses. These deliberations confirmed the necessity of developing an oral formulation of FBZ that could provide high efficacy in a short regimen. Although the toxicologic data developed for FBZ as part of this program were of concern, the potential to achieve cure with short-term and infrequent exposure meant that the risk–benefit ratio could be favorable, and JRD elected to continue its formulation development and preclinical studies. However, although parenteral formulations of FBZ routinely achieve 100% efficacy in animal models, short dosing regimens using the oral formulation did not consistently achieve desired cure rates in multiple animal models. Given the toxicological profile of FBZ and the incompatability of injectable formulations for filariasis control programs, it was concluded that parenteral FBZ should not be developed as an alternative to the oral formulation. In early 2017, after assembling all relevant preclinical data, JRD determined that the risk–benefit ratio for FBZ as a macrofilaricide was insufficient to justify continuing the program.

It is important to stress that the original goal of the project was met: formulations that afford high oral bioavailability of FBZ were generated and provided sufficient efficacy in a suitable regimen to warrant consideration for further development. However, oral regimens associated with efficacy also induced adverse events in animal studies that were of sufficient severity to preclude first-in-human studies. This may be related, at least in part, to species-dependent generation of the reduced metabolite of the drug [22, 30], which led to unanticipated toxicological effects [19, 20, 22, 31].

The effort to evaluate FBZ as a candidate macrofilaricide illustrates the benefit of repurposing already licensed drugs for new neglected tropical disease indications in a data-driven process with clear objectives. The contributions of JRD led to a rational decision to suspend development of FBZ for this indication due to unanticipated toxicological results, but the data show that appropriate exposure to a benzimidazole anthelmintic can achieve macrofilaricidal efficacy after oral administration.
